# Modeling traumatic brain injury lifetime data: Improved estimators for the Generalized Gamma distribution under small samples

**DOI:** 10.1371/journal.pone.0221332

**Published:** 2019-08-30

**Authors:** Pedro L. Ramos, Diego C. Nascimento, Paulo H. Ferreira, Karina T. Weber, Taiza E. G. Santos, Francisco Louzada

**Affiliations:** 1 Institute of Mathematics and Computer Sciences, University of São Paulo, São Carlos, Brazil; 2 Institute of Mathematics and Statistics, Federal University of Bahia, Salvador, Brazil; 3 Medical School of Ribeirão Preto, University of São Paulo, Ribeirão Preto, Brazil; Tongii University, CHINA

## Abstract

In this paper, from the practical point of view, we focus on modeling traumatic brain injury data considering different stages of hospitalization, related to patients’ survival rates following traumatic brain injury caused by traffic accidents. From the statistical point of view, the primary objective is related to overcoming the limited number of traumatic brain injury patients available for studying by considering different estimation methods to obtain improved estimators of the model parameters, which can be recommended to be used in the presence of small samples. To have a general methodology, at least in principle, we consider the very flexible Generalized Gamma distribution. We compare various estimation methods using extensive numerical simulations. The results reveal that the penalized maximum likelihood estimators have the smallest mean square errors and biases, proving to be the most efficient method among the investigated ones, mainly to be used in the presence of small samples. The Simulated Annealing technique is used to avoid numerical problems during the optimization process, as well as the need for good initial values. Overall, we considered an amount of three real data sets related to traumatic brain injury caused by traffic accidents to demonstrate that the Generalized Gamma distribution is a simple alternative to be used in this type of applications for different occurrence rates and risks, and in the presence of small samples.

## 1 Introduction

Gamma distribution plays an important role in statistics as one of the most used generalizations of the Exponential distribution due to its various special cases (such as Exponential and Chi-square). This distribution has been used in different scenarios, such as reliability engineering, environmental modeling, and health research, to list a few (see Louzada and Ramos [1] and the references therein). Stacy [2] proposed an important generalization of the Gamma distribution to unify other relevant distributions, e.g., Weibull and Lognormal. This generalization, called Generalized Gamma (GG) distribution, has been successfully applied in diverse areas, such as reliability, data processing, and meteorology, among others (see Cox et al. [3] and the references therein). Moreover, it keeps the characteristic of incorporating only the support of a positive random variable *T*, and its probability density function (pdf) is given by

$$\begin{array}{c} f\left(t|\phi ,\mu ,\alpha \right)=\frac{\alpha }{\Gamma (\phi )}\mu^{\alpha \phi }t^{\alpha \phi -1}\;\text{exp}\;\left\{-{\left(\mu t\right)}^{\alpha }\right\}, \end{array}$$ (1)

where *t* > 0, $\Gamma \left(\phi \right)=\int_{0}^{\infty }e^{-t}t^{\phi -1}dt$ is the gamma function, *α* > 0 and *ϕ* > 0 are the shape parameters and *μ* > 0 is the scale parameter. The GG distribution includes various sub-models as special cases, such as the Log-Normal, Weibull, Gamma, Half-Normal, Nakagami-m, Rayleigh, Maxwell-Boltzmann, and Chi distributions.

Frequentist inference for the GG distribution has been widely considered in the literature. Stacy and Mihram [4] derived the maximum likelihood estimators (MLEs). However, Harger and Bain [5] later showed that the nonlinear equations obtained by the maximum likelihood approach are unstable. DiCiccio [6] discussed approximate conditional inference methods for this distribution. Huang and Hwang [7] used the method of moments to perform inference for the GG distribution. Furthermore, Khodabin and Ahmadabadi [8] compared the method of moment estimators and MLEs, whose results revealed that most of the time the MLEs showed greater performance even though under the presence of estimation limitations. Recently, Noufaily and Jones [9] discussed some different approaches to maximize the likelihood function; the proposed numerical technique returned smaller proportions of errors during the maximization process, but still failed in a significant number of samples, which is undesirable.

### 1.1 Overview of the TBI problematic

Trauma is a multisystem health condition that represents the third cause of death worldwide, surpassed only by cerebrovascular diseases and cancer [10]. It is estimated that over sixty million people have trauma each year, and nearly 16,000 people die every day after some traumatic injury. Traumatic Brain Injury (TBI) represents one of the significant causes of death and disability among the trauma epidemiology data [10, 11]. Most of the patients with TBI are young, economically active adults and more likely to have been involved in a traffic accident [12–16]. Therefore, TBI is considered a public health concern that leads to high costs of hospitalization with various economic and social burdens.

The limited available data in the TBI problematic, usually presented in a small number of patients, motivated the current study, which relied on a data set of a longitudinal observational investigation of patients after TBI due to traffic accidents admitted to a Brazilian Emergency Department. Investigating optimal statistical analyses in this population is essential for providing impactful information applied not only to patients but also to their families, caregivers, and society in general [17].

### 1.2 Current statistical methods and their limitations

In the literature, there are various classical methods for estimating the unknown parameters of probability distributions. Under the frequentist approach, the primary interest is to compare the maximum likelihood estimation method with other estimation procedures. Related studies about different distributions have also been presented in the literature [18–22]. In this work, we consider several of these estimation procedures, such as least squares, the weighted least squares, the maximum product of spacings, and the Anderson-Darling maximum goodness-of-fit estimators.

An intensive simulation study is conducted to compare these estimation methods. However, we observe two significant problems. The first is that, for some methods, the estimation procedures fail in finding the target estimates, i.e., report convergence problems in the maximization/minimization process. The second is related to the occurrence of a significant bias in the obtained estimates for small samples. In order to overcome this problem, we propose a penalized maximum likelihood estimator, which, combined with a very useful practical algorithm called Simulated Annealing (SANN) (for further details, see Kirkpatrick et al. [23]), guarantees the best convergence not depending on the conditions of the problem (i.e., the initial values), even when it has several local extrema. Prentice [24] argued that the approximately normal distribution for *ϕ*, using the maximum likelihood theory, may not be achieved even for sample sizes equal to or larger than 400. Due to the asymptotic relationship of the maximum likelihood estimator with the penalized maximum likelihood estimator, the same problem may be observed. Therefore, we consider a bootstrap approach to building accurate confidence intervals (see DiCiccio and Efron [25]) for small and moderate samples. Finally, by combining all these approaches, one can perform inference for the flexible GG distribution with good precision even for small sample sizes.

The paper is organized as follows. Section 2 presents some properties of the GG distribution, including its cumulative distribution, survival and hazard functions, and its moments. Additionally, the SANN algorithm is also discussed in detail, and implementation procedures are presented. Section 3 discusses the eight estimation methods considered in this paper. Section 4 shows a simulation study, using synthetic data, designed to identify the most efficient estimation procedure. In Section 5, we apply our proposed methodology to three new real data sets provided by a medical school, which contain the TBI patients’ lifetime risk among different hospitalization stages. Finally, some final comments are given in Section 6.

## 2 Background

Let *T* be a random variable with GG distribution, i.e. *T* ~ GG (*ϕ*, *μ*, *α*). Then, its cumulative distribution function (cdf) is given by

$$\begin{array}{c} F\left(t|\phi ,\mu ,\alpha \right)=\int_{0}^{{\left(\mu t\right)}^{\alpha }}\frac{1}{\Gamma (\phi )}w^{\phi -1}e^{-w}dw=\frac{\gamma (\phi ,{\left(\mu t\right)}^{\alpha })}{\Gamma (\phi )}\;, \end{array}$$ (2)

where $\gamma \left(y,x\right)=\int_{0}^{x}w^{y-1}e^{-w}dw$ is called lower incomplete gamma function. The survival function is given by

$$\begin{array}{c} S\left(t|\phi ,\mu ,\alpha \right)=1-F\left(t|\phi ,\mu ,\alpha \right)=1-\int_{0}^{{\left(\mu t\right)}^{\alpha }}\frac{1}{\Gamma (\phi )}w^{\phi -1}e^{-w}dw=\frac{\Gamma (\phi ,{\left(\mu t\right)}^{\alpha })}{\Gamma (\phi )}\;, \end{array}$$ (3)

where $\Gamma \left(y,x\right)=\int_{x}^{\infty }w^{y-1}e^{-w}dw$ is the upper incomplete gamma function. The lower and upper incomplete gamma functions are standard functions in many pieces of software, such as R, SAS and Ox. Finally, the hazard function is given by

$$\begin{array}{c} h\left(t|\phi ,\mu ,\alpha \right)=\frac{f(t|\phi ,\mu ,\alpha )}{S(t|\phi ,\mu ,\alpha )}=\frac{\alpha \mu^{\alpha \phi }t^{\alpha \phi -1}\;\text{exp}\;\{-{\left(\mu t\right)}^{\alpha }\}}{\Gamma (\phi ,{\left(\mu t\right)}^{\alpha })}. \end{array}$$ (4)

**Fig 1:**
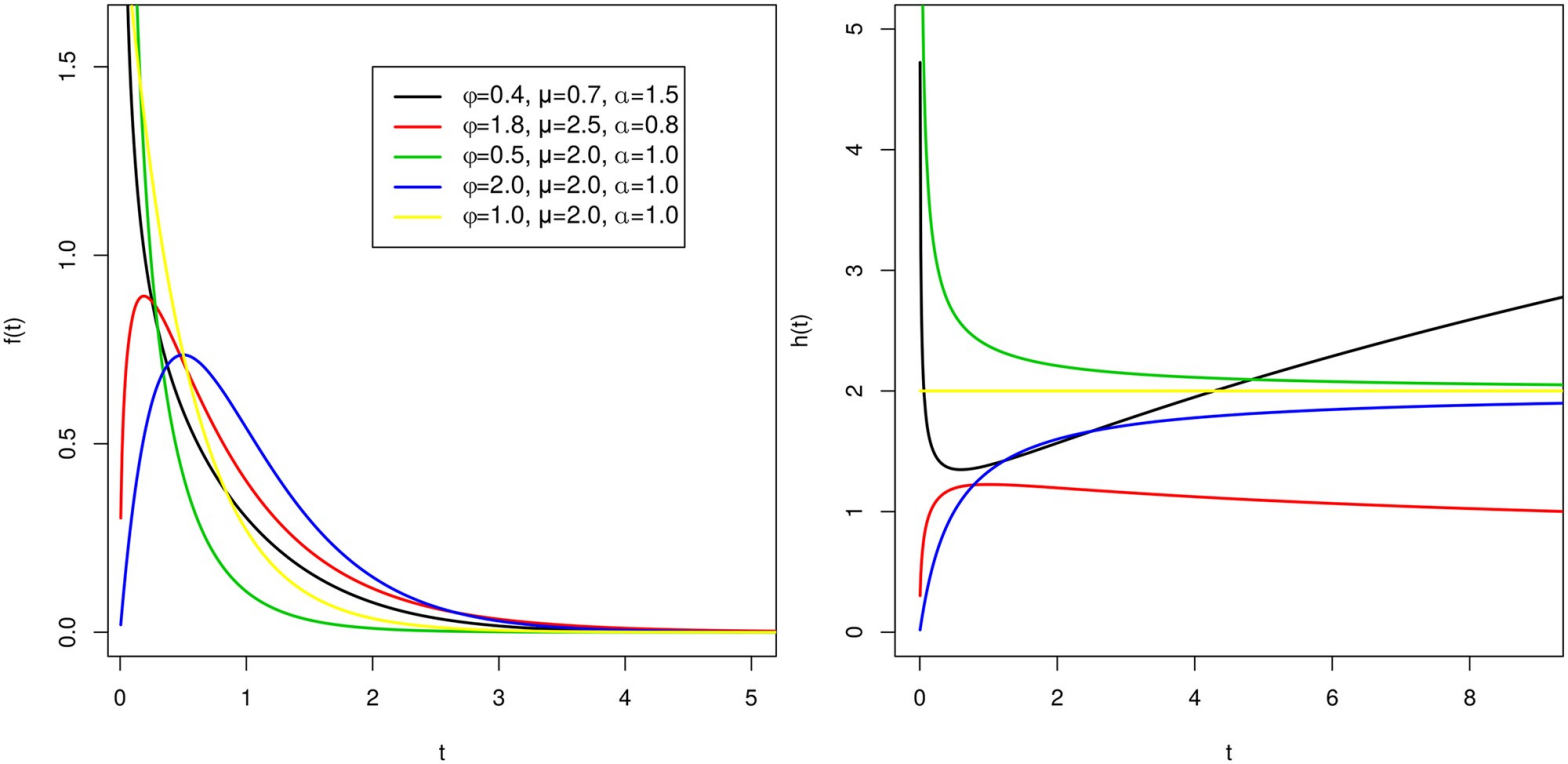
Shapes of the pdf and hazard function. (A) Pdf of the GG distribution. (B) Hazard function of the GG distribution.

Glaser [26] showed that the hazard function (4) of the GG distribution can capture basic shapes, such as constant, increasing, decreasing, bathtub and unimodal. Fig 1 presents some examples of the shapes of the pdf and hazard function, considering different values of *ϕ*, *μ* and *α*.

The *r*-th moment of *T* about the origin can be obtained by

$$\begin{array}{c} E[T^{r}]=\frac{\Gamma (\phi +\frac{r}{\alpha })}{\mu^{r}\Gamma \left(\phi \right)}. \end{array}$$ (5)

Then, the mean and variance of the GG distribution are given, respectively, by

$$\begin{array}{c} E[T]=\frac{\Gamma (\phi +\frac{1}{\alpha })}{\mu \Gamma (\phi )}\;\;\text{and}\;\;\text{Var}[T]=\frac{1}{\mu^{2}}[\frac{\Gamma (\phi +\frac{2}{\alpha })}{\Gamma (\phi )}-(\frac{\Gamma (\phi +\frac{1}{\alpha })}{\Gamma (\phi )}{)}^{2}]. \end{array}$$ (6)

### 2.1 Simulated annealing algorithm

The SANN algorithm was developed via the generalization of the Metropolis algorithm (Metropolis et al. [27]) to simulate the changes in the energy of molten metal when lowering its temperature slowly. The purpose of the cooling process is to reach a globally minimum energy state, i.e., to obtain a solid that is in its ground state. However, as pointed out by Salter and Pearl [28], if the temperature is lowered too fast, the resulting solid can become trapped in a metastable state that is not its ground state. Some authors, such as Kirkpatrick et al. [23], noticed the analogy between the cooling process of a substance to its minimum energy state and the minimization of a function by using a stochastic search strategy. In this case, the metastable state represents a local minimum, the ground state represents the global minimum, and the cooling rate corresponds to some parameters that control the possible solutions by the search algorithm.

Let $g:R^{d}\to R$ be the function to be minimized (objective function), let $x_{0}=(x_{1}^{\left(0\right)},\ldots ,x_{d}^{\left(0\right)})$ be the initial solution (initial points), and let *k*_0_ be the initial value of the control parameter (initial temperature). The SANN algorithm can be described in a formal and general way as follows.

1. Set *i* = 0.
2. From the current solution, ***x***_*i*_, generate a potential solution, ***x***_*j*_, according to a specific generation scheme.
3. If Δ*g* = *g*(***x***_*j*_) − *g*(***x***_*i*_) ≤ 0, then set ***x***_*i*+1_ = ***x***_*j*_ with probability *p* = 1. Otherwise, set ***x***_*i*+1_ = ***x***_*j*_ with probability *p* = exp {− Δ*g* / *k*_*i*_}.
4. Update the value of the control parameter, *k*_*i*_, and set *i* = *i* + 1. Then, go to step 2.

Steps 2-4 are repeated, e.g., until the value of the control parameter, *k*_*i*_, is sufficiently small, or the same solution is repeatedly generated in many successive iterations.

Next, some useful remarks about the above algorithm are given.

1. For some choices of *k*_0_, including *k*_0_ = log (*g* (***x***_0_)), see, e.g., Aarts and Korst [29].
2. In step 2, the potential solutions, ***x***_*j*_’s, are randomly chosen within a range. For instance, through ***x***_*j*_ = ***x***_*j*_ + *r****V***, where *r* is a uniformly distributed random number in the interval (−1, 1), i.e. *r* ~ U(−1, 1), and ***V*** is a vector (of length *d*) of step sizes. After *s* iterations, the SANN adjusts its search bounds for each variable so that 50% of all moves will be accepted, either enlarging these bounds to select a new ground to move to or shrinking them to a minimum.
3. In step 3, for the cases where Δ*g* > 0, we generate *u* ~ U(0, 1) and move to ***x***_*j*_ only if *u* < *p*. Thus, accepting worse solutions may prevent the process from becoming stuck at local minima.
4. In step 4, after *m* iterations, temperature (control parameter) *k* drops as *k*′ = *r*_*k*_ × *k*, where 0 ≤ *r*_*k*_ ≤ 1 is the rate of temperature reduction given the initial annealing/cooling schedule. Usually, *r*_*k*_ = 0.95.
5. As pointed out by Salter and Pearl [28], by the Markov chain theory, the SANN algorithm can be shown to converge to a stationary distribution for which the set of optimal solutions has probability 1, under certain conditions of both the sequence of control parameters and the generation scheme (see, e.g., Aarts and Korst [29], Haario and Saksman [30]).

Although presented above as a minimization problem, the SANN algorithm can be easily modified/adapted to cases where the interest resides on maximizing the function *g*(⋅).

Several variants of the SANN algorithm have been proposed in the recent literature. Among them, we can mention the relevant works of Torres-Jimenez and Rodriguez-Tello [31], Torres-Jimenez et al. [32], and Izquierdo-Marquez et al. [33].

## 3 Inference

In this section, we present different frequentist estimation methods to obtain the estimates for the parameters *ϕ*, *μ*, and *α* of the GG distribution.

### 3.1 Common estimators

The method of moments (MM) is one of the oldest procedures used for estimating parameters in statistical models. It is still widely used mainly because of its simplicity. For instance, for the two-parameter Gamma distribution, MM estimators have closed-form expressions.

Huang and Hwang [7] derived the MM estimators for the GG distribution, pointing out the need for solving two nonlinear equations, to find such estimators. Let *t*_1_, …, *t*_*n*_ be a random sample of size *n* from *T* ∼ GG(*ϕ*, *μ*, *α*). The moments estimators are obtained by solving

$$\begin{array}{c} \bar{t}-\frac{\Gamma (\phi +\frac{1}{\alpha })}{\mu \Gamma (\phi )}=0\;\;\;\text{and}\;\;\;\frac{s^{2}}{n{\bar{t}}^{2}}-\frac{\Gamma \left(\phi \right)\Gamma (\phi +\frac{2}{\alpha })-[\Gamma (\phi +\frac{1}{\alpha }){]}^{2}}{\Gamma \left(\phi \right)\Gamma (\phi +\frac{2}{\alpha })+\left(n-1\right)[\Gamma (\phi +\frac{1}{\alpha }){]}^{2}}=0, \end{array}$$ (7)

where $\bar{t}=\frac{1}{n}\sum_{i=1}^{n}t_{i}$ and $s=\sqrt{\frac{1}{n-1}\sum_{i=1}^{n}(t_{i}-\bar{t}{)}^{2}}$ are the sample mean and standard deviation, respectively; while the estimate of *ϕ* can be obtained by

$$\begin{array}{c} {\hat{\phi }}_{\text{MM}}=\frac{1}{n}\sum_{i=1}^{n}{\left({\hat{\mu }}_{\text{MM}}t_{i}\right)}^{{\hat{\alpha }}_{\text{MM}}}, \end{array}$$ (8)

where ${\hat{\mu }}_{\text{MM}}$ and ${\hat{\alpha }}_{\text{MM}}$ are obtained by solving the nonlinear equations in (7). The MM estimators of all GG model parameters do not have closed-form expressions, which is undesirable. Another disadvantage of this approach is that the authors did not discuss the asymptotic properties of the MM estimators. Therefore, no interval estimates for *ϕ*, *μ* and *α* can be constructed without further research.

Another common procedure is to consider the ordinary least squares (OLS) estimators. The ${\hat{\phi }}_{\text{OLS}}$, ${\hat{\mu }}_{\text{OLS}}$ and ${\hat{\alpha }}_{\text{OLS}}$ estimates can be obtained by minimizing, with respect to *ϕ*, *μ* and *α*, the following equation:

$$\begin{array}{c} V(\phi ,\mu ,\alpha )=\sum_{i=1}^{n}[\frac{\gamma (\phi ,{\left(\mu t_{\left(i\right)}\right)}^{\alpha })}{\Gamma (\phi )}-\frac{i}{n+1}{]}^{2}, \end{array}$$ (9)

where *t*_(1)_ ≤ *t*_(2)_ ≤ ⋯ ≤ *t*_(*n*)_ are the order statistics of a random sample of size *n*. Equivalently, these estimates can be obtained by solving the nonlinear equations:

$$\begin{array}{c} \begin{array}{cc} & \sum_{i=1}^{n}[\frac{\gamma (\phi ,{\left(\mu t_{\left(i\right)}\right)}^{\alpha })}{\Gamma (\phi )}-\frac{i}{n+1}]\Delta_{j}(t_{\left(i\right)}|\phi ,\mu ,\alpha )=0,\;\;\;\text{for}\;\;\;j=1,2,3, \end{array} \end{array}$$ (10)

where

$$\begin{array}{c} \begin{array}{cc} \Delta_{1}(t_{\left(i\right)}|\phi ,\mu ,\alpha )= & \frac{\partial }{\partial \phi }F(t_{\left(i\right)}|\phi ,\mu ,\alpha ),\;\Delta_{2}(t_{\left(i\right)}|\phi ,\mu ,\alpha )=\frac{\alpha {\left(\mu t_{\left(i\right)}\right)}^{\phi \alpha }e^{-{\left(\mu t_{\left(i\right)}\right)}^{\alpha }}}{\mu \Gamma (\phi )},\\ & \Delta_{3}(t_{\left(i\right)}|\phi ,\mu ,\alpha )=\frac{\text{log}\;\left(\mu t_{\left(i\right)}\right){\left(\mu t_{\left(i\right)}\right)}^{\phi \alpha }e^{-{\left(\mu t_{\left(i\right)}\right)}^{\alpha }}}{\Gamma (\phi )}. \end{array} \end{array}$$ (11)

Note that the solution of Δ_1_(*t*_(*i*)_|*ϕ*, *μ*, *α*) involves a non-trivial partial derivative of the lower incomplete gamma function. However, this can be easily achieved numerically with high precision.

The weighted least squares (WLS) estimators, ${\hat{\phi }}_{\text{WLS}}$, ${\hat{\mu }}_{\text{WLS}}$ and ${\hat{\alpha }}_{\text{WLS}}$, can be obtained by minimizing

$$\begin{array}{c} W(\phi ,\mu ,\alpha )=\sum_{i=1}^{n}\frac{\left(n+1{)}^{2}(n+2\right)}{i(n-i+1)}[\frac{\gamma (\phi ,{\left(\mu t_{\left(i\right)}\right)}^{\alpha })}{\Gamma (\phi )}-\frac{i}{n+1}{]}^{2} \end{array}$$ (12)

with respect to *ϕ*, *μ* and *α*. These estimates can also be obtained by solving the nonlinear equations:

$$\begin{array}{c} \sum_{i=1}^{n}\frac{\left(n+1{)}^{2}(n+2\right)}{i(n-i+1)}[\frac{\gamma (\phi ,{\left(\mu t_{\left(i\right)}\right)}^{\alpha })}{\Gamma (\phi )}-\frac{i}{n+1}]\Delta_{j}(t_{\left(i\right)}|\phi ,\mu ,\alpha )=0, \end{array}$$ (13)

for *j* = 1, 2, 3.

### 3.2 Maximum likelihood estimators

The maximum likelihood (ML) estimation method is widely used for the GG distribution due to the invariance and asymptotic properties of the obtained estimators. Let ***t*** = (*t*_1_,…, *t*_*n*_)′ be a random sample of size *n* from a GG(*ϕ*, *μ*, *α*) population. Then, the likelihood function of (1) is given by

$$\begin{array}{c} L\left(\phi ,\mu ,\alpha |t\right)=\frac{\alpha^{n}}{{\left[\Gamma \left(\phi \right)\right]}^{n}}\mu^{n\alpha \phi }(\prod_{i=1}^{n}t_{i}^{\alpha \phi -1})\;\text{exp}\;\{-\mu^{\alpha }\sum_{i=1}^{n}t_{i}^{\alpha }\}. \end{array}$$ (14)

The log-likelihood function of (14) is given by

$$\begin{array}{c} \ell \left(\phi ,\mu ,\alpha |t\right)=n\;\text{log}\;\left(\alpha \right)-n\;\text{log}\;(\Gamma (\phi ))+n\alpha \phi \;\text{log}\;\left(\mu \right)+(\alpha \phi -1)\sum_{i=1}^{n}\;\text{log}\;(t_{i})-\mu^{\alpha }\sum_{i=1}^{n}t_{i}^{\alpha }. \end{array}$$ (15)

By solving the expressions: $\frac{\partial }{\partial \phi }\ell \left(\phi ,\mu ,\alpha |t\right)=0$, $\frac{\partial }{\partial \mu }\ell \left(\phi ,\mu ,\alpha |t\right)=0$ and $\frac{\partial }{\partial \alpha }\ell \left(\phi ,\mu ,\alpha |t\right)=0$, the following nonlinear equations can be obtained, respectively:

$$\begin{array}{c} n\hat{\alpha }\;\text{log}\;\left(\hat{\mu }\right)+\hat{\alpha }\sum_{i=1}^{n}\;\text{log}\;\left(t_{i}\right)=n\psi \left(\hat{\phi }\right), \end{array}$$ (16)

$$\begin{array}{c} n\hat{\alpha }\hat{\phi }=\hat{\alpha }{\hat{\mu }}^{\hat{\alpha }}\sum_{i=1}^{n}{t_{i}}^{\hat{\alpha }}\;\;\;\text{and} \end{array}$$ (17)

$$\begin{array}{c} \frac{n}{\hat{\alpha }}+n\hat{\phi }\;\text{log}\;\left(\hat{\mu }\right)+\hat{\phi }\sum_{i=1}^{n}\;\text{log}\;\left(t_{i}\right)={\hat{\mu }}^{\hat{\alpha }}\sum_{i=1}^{n}{t_{i}}^{\hat{\alpha }}\;\text{log}\;\left(\hat{\mu }t_{i}\right), \end{array}$$ (18)

where $\psi \left(k\right)=\frac{\Gamma^{\prime }\left(k\right)}{\Gamma (k)}$ is the digamma function. The solutions of the above Eq (17) yield the MLEs. After some algebraic manipulations, we have

$$\begin{array}{c} \hat{\mu }=(\frac{n\hat{\phi }}{\sum_{i=1}^{n}t_{i}^{\hat{\alpha }}}{)}^{\frac{1}{\hat{\alpha }}}, \end{array}$$ (19)

$$\begin{array}{c} \hat{\phi }=\frac{\sum_{i=1}^{n}t_{i}^{\hat{\alpha }}}{n\sum_{i=1}^{n}t_{i}^{\hat{\alpha }}\;\text{log}\;\left(t_{i}^{\hat{\alpha }}\right)-\sum_{i=1}^{n}t_{i}^{\hat{\alpha }}\sum_{i=1}^{n}\;\text{log}\;\left(t_{i}^{\hat{\alpha }}\right)} \end{array}$$ (20)

and the MLE of *α* is obtained by solving the nonlinear equation:

$$\begin{array}{c} h\left(\alpha \right)=n\psi \left(n\hat{\phi }\right)+\alpha \sum_{i=1}^{n}\;\text{log}\;\left(t_{i}\right)-\frac{1}{\hat{\phi }}-\psi \left(\hat{\phi }\right)-\text{log}\;(\sum_{i=1}^{n}t_{i}^{\alpha })=0. \end{array}$$ (21)

Although only one nonlinear equation has to be solved, there are usually different local maxima, which lead to different estimates than expected. On the other hand, under mild conditions, the MLEs are asymptotically normally distributed with a joint trivariate normal distribution given by

$$\begin{array}{c} \left(\hat{\phi },\hat{\mu },\hat{\alpha }\right)∼N_{3}(\left(\phi ,\mu ,\alpha \right),I^{-1}\left(\phi ,\mu ,\alpha \right))\;\;\text{for}\;\;n\to \infty , \end{array}$$ (22)

where *I*(*ϕ*, *μ*, *α*) is the Fisher information matrix (see Hager and Bain [5] for a detailed discussion) given by

$$\begin{array}{c} I\left(\phi ,\mu ,\alpha \right)=n[\begin{array}{ccc} \psi^{{}^{\prime }}\left(\phi \right) & \frac{\alpha }{\mu } & -\frac{\psi (\phi )}{\alpha }\\ \frac{\alpha }{\mu } & \frac{\phi \alpha^{2}}{\mu^{2}} & -\frac{1+\phi \psi (\phi )}{\mu }\\ -\frac{\psi (\phi )}{\alpha } & -\frac{1+\phi \psi (\phi )}{\mu } & \frac{1+2\psi \left(\phi \right)+\phi \psi^{{}^{\prime }}\left(\phi \right)+\phi {\left[\psi \left(\phi \right)\right]}^{2}}{\alpha^{2}} \end{array}] \end{array}$$ (23)

and $\psi^{\prime }\left(k\right)=\frac{\partial }{\partial k}\psi \left(k\right)$ is the trigamma function.

### 3.3 Penalized maximum likelihood estimators

Firth [34] proved that the bias of MLEs can be reduced by considering a penalization in the likelihood function. Moreover, the author showed that in exponential families with canonical parameterization, the first-order term is removed by using the Jeffreys prior [35] as a penalization term. The Jeffreys prior for the GG distribution is computed by |*I*(*ϕ*, *μ*, *α*)|^1/2^, where |⋅| stands for the determinant of the Fisher information matrix (23), which results in

$$\begin{array}{c} \pi_{J}(\phi ,\mu ,\alpha )\propto \frac{\sqrt{\phi^{2}{\left[\psi^{{}^{\prime }}\left(\phi \right)\right]}^{2}-\psi^{{}^{\prime }}\left(\phi \right)-1}}{\mu }. \end{array}$$ (24)

As previously stated, the first-order term related to the bias is removed in the case of distributions that belong to the exponential family. On the other hand, the GG distribution is not a member of the exponential family. However, this penalization also allows us to improve the estimates, even not ensuring that the improvement is of the first order. Note that when *ϕ* = 1, the GG distribution reduces to the Weibull distribution, for which the Jeffreys prior is given by *π*_J_ (*μ*, *α*) ∝ (*μα*)^−1^. The extra *α*^−1^ helps us to decrease the bias of *α*; therefore, since *π*_J_ (*ϕ*, *μ*, *α*) is not a function of *α*, we consider the following penalization:

$$\begin{array}{c} \pi (\phi ,\mu ,\alpha )\propto \frac{\sqrt{\phi^{2}{\left[\psi^{{}^{\prime }}\left(\phi \right)\right]}^{2}-\psi^{{}^{\prime }}\left(\phi \right)-1}}{\mu \alpha }. \end{array}$$ (25)

The penalized likelihood function of *ϕ*, *μ* and *α*, using the Jeffreys prior (25), is given by

$$\begin{array}{c} \begin{array}{cc} L_{P}\left(\phi ,\mu ,\alpha |t\right)= & \frac{\alpha^{n-1}\sqrt{\phi^{2}{\left[\psi^{{}^{\prime }}\left(\phi \right)\right]}^{2}-\psi^{{}^{\prime }}\left(\phi \right)-1}}{{\left[\Gamma \left(\phi \right)\right]}^{n}}\mu^{n\alpha \phi -1}(\prod_{i=1}^{n}t_{i}^{\alpha \phi -1})\;\text{exp}\;\{-\mu^{\alpha }\sum_{i=1}^{n}t_{i}^{\alpha }\}. \end{array} \end{array}$$ (26)

The log-likelihood function of (26) is given by

$$\begin{array}{c} \begin{array}{cc} \ell_{P}\left(\phi ,\mu ,\alpha |t\right)= & \;\left(n-1\right)\;\text{log}\;\left(\alpha \right)+\frac{1}{2}\;\text{log}\;(\phi^{2}{\left[\psi^{{}^{\prime }}\left(\phi \right)\right]}^{2}-\psi^{{}^{\prime }}\left(\phi \right)-1)+\left(n\alpha \phi -1\right)\;\text{log}\;\left(\mu \right)\\ & +(\alpha \phi -1)\sum_{i=1}^{n}\;\text{log}\;(t_{i})-\mu^{\alpha }\sum_{i=1}^{n}t_{i}^{\alpha }-n\;\text{log}\;(\Gamma (\phi )). \end{array} \end{array}$$ (27)

By solving the expressions: $\frac{\partial }{\partial \phi }\ell_{P}\left(\phi ,\mu ,\alpha |t\right)=0$, $\frac{\partial }{\partial \mu }\ell_{P}\left(\phi ,\mu ,\alpha |t\right)=0$ and $\frac{\partial }{\partial \alpha }\ell_{P}\left(\phi ,\mu ,\alpha |t\right)=0$, the following nonlinear equations can be obtained, respectively:

$$\begin{array}{c} \frac{{\hat{\phi }}^{2}\psi^{{}^{\prime }}\left(\hat{\phi }\right)\psi^{{}^{\prime \prime }}\left(\hat{\phi }\right)+\hat{\phi }{\left[\psi^{{}^{\prime }}\left(\hat{\phi }\right)\right]}^{2}-0.5\psi^{{}^{\prime \prime }}\left(\hat{\phi }\right)}{{\hat{\phi }}^{2}{\left[\psi^{{}^{\prime }}\left(\hat{\phi }\right)\right]}^{2}-\psi^{{}^{\prime }}\left(\hat{\phi }\right)-1}+n\hat{\alpha }\;\text{log}\;\left(\hat{\mu }\right)=n\psi \left(\hat{\phi }\right)-\hat{\alpha }\sum_{i=1}^{n}\;\text{log}\;\left(t_{i}\right), \end{array}$$ (28)

$$\begin{array}{c} n\hat{\alpha }\hat{\phi }-1=\hat{\alpha }{\hat{\mu }}^{\hat{\alpha }}\sum_{i=1}^{n}{t_{i}}^{\hat{\alpha }}\;\;\;\text{and} \end{array}$$ (29)

$$\begin{array}{c} \frac{n-1}{\hat{\alpha }}+n\hat{\phi }\;\text{log}\;\left(\hat{\mu }\right)+\hat{\phi }\sum_{i=1}^{n}\;\text{log}\;\left(t_{i}\right)={\hat{\mu }}^{\hat{\alpha }}\sum_{i=1}^{n}{t_{i}}^{\hat{\alpha }}\;\text{log}\;\left(\hat{\mu }t_{i}\right). \end{array}$$ (30)

Note that one of the parameters can be isolated, in order to obtain two nonlinear equations. The three possible expressions are given by

$$\begin{array}{c} \hat{\mu }=(\frac{n\hat{\alpha }\hat{\phi }-1}{\hat{\alpha }\sum_{i=1}^{n}{t_{i}}^{\hat{\alpha }}}{)}^{\frac{1}{\hat{\alpha }}}, \end{array}$$ (31)

$$\begin{array}{c} \hat{\phi }=\frac{\hat{\alpha }{\hat{\mu }}^{\hat{\alpha }}\sum_{i=1}^{n}{t_{i}}^{\hat{\alpha }}+1}{n\hat{\alpha }}, \end{array}$$ (32)

$$\begin{array}{c} \hat{\alpha }=\frac{n\psi^{\prime }\left(\hat{\phi }\right)({\hat{\phi }}^{2}{\left[\psi^{{}^{\prime }}\left(\hat{\phi }\right)\right]}^{2}-\psi^{{}^{\prime }}\left(\hat{\phi }\right)-1)-{\hat{\phi }}^{2}\psi^{{}^{\prime }}\left(\hat{\phi }\right)\psi^{{}^{\prime \prime }}\left(\hat{\phi }\right)+\hat{\phi }{\left[\psi^{{}^{\prime }}\left(\hat{\phi }\right)\right]}^{2}-0.5\psi^{{}^{\prime \prime }}\left(\hat{\phi }\right)}{\left(n\;\text{log}\;\left(\hat{\mu }\right)+\sum_{i=1}^{n}\text{log}\left(t_{i}\right))({\hat{\phi }}^{2}{\left[\psi^{{}^{\prime }}\left(\hat{\phi }\right)\right]}^{2}-\psi^{{}^{\prime }}\left(\hat{\phi }\right)-1\right)}. \end{array}$$ (33)

From Eqs (31)–(33), we observe that, considering (32), the penalized maximum likelihood (PML) estimators are achieved with less computational effort. Therefore, we will consider the nonlinear Eqs (28) and (30), where $\hat{\phi }$ is obtained from (32).

Although Firth [34] proved that the PML estimators obtained from the penalized likelihood or log-likelihood function in the exponential family of distributions are always finite and, in addition, always exist, the same cannot be done for the GG distribution. For this model, the MLEs can have no solution or several solutions (see Wingo [36]). This problem is observed computationally, since it is very complex to prove analytically. It is also complicated to demonstrate analytically the results for the PML estimators. Note that our main goals here are to propose a method to circumvent these computation difficulties, and achieve improved estimates for the parameters.

The Fisher information matrix *I*_P_ (*ϕ*, *μ*, *α*) is given by

$$\begin{array}{c} I_{P}\left(\phi ,\mu ,\alpha \right)=n[\begin{array}{ccc} \frac{I_{P}\left(\phi \right)}{n}+\psi^{{}^{\prime }}\left(\phi \right) & \frac{\alpha }{\mu } & -\frac{\psi (\phi )}{\alpha }\\ \frac{\alpha }{\mu } & \frac{\phi \alpha^{2}}{\mu^{2}} & -\frac{1+\phi \psi (\phi )}{\mu }\\ -\frac{\psi (\phi )}{\alpha } & -\frac{1+\phi \psi (\phi )}{\mu } & \frac{1+2\psi \left(\phi \right)+\phi \psi^{{}^{\prime }}\left(\phi \right)+\phi {\left[\psi \left(\phi \right)\right]}^{2}-n^{-1}}{\alpha^{2}} \end{array}], \end{array}$$ (34)

where

$$\begin{array}{c} \begin{array}{cc} I_{P}\left(\phi \right)= & \frac{\left(\phi^{2}\psi^{{}^{\prime }}\left(\phi \right)\psi^{{}^{\prime \prime }}\left(\phi \right)+\phi {\left[\psi^{{}^{\prime }}\left(\phi \right)\right]}^{2}-0.5\psi^{{}^{\prime \prime }}\left(\phi \right))(2\phi^{2}\psi^{{}^{\prime }}\left(\phi \right)\psi^{{}^{\prime \prime }}\left(\phi \right)+2\phi {\left[\psi^{{}^{\prime }}\left(\phi \right)\right]}^{2}-\psi^{{}^{\prime \prime }}\left(\phi \right)\right)}{{\left(\phi^{2}{\left[\psi^{{}^{\prime }}\left(\phi \right)\right]}^{2}-\psi^{{}^{\prime }}\left(\phi \right)-1\right)}^{2}}\\ & -\frac{\phi^{2}{\left[\psi^{{}^{\prime \prime }}\left(\phi \right)\right]}^{2}+\phi^{2}\psi^{{}^{\prime }}\left(\phi \right)\psi^{{}^{\prime \prime \prime }}\left(\phi \right)+4\phi \psi^{{}^{\prime }}\left(\phi \right)+{\left[\psi^{{}^{\prime }}\left(\phi \right)\right]}^{2}-0.5\psi^{{}^{\prime \prime \prime }}\left(\phi \right)}{\phi^{2}{\left[\psi^{{}^{\prime }}\left(\phi \right)\right]}^{2}-\psi^{{}^{\prime }}\left(\phi \right)-1}. \end{array} \end{array}$$ (35)

It can be easily noted that *I*_P_(*ϕ*) → *I*(*ϕ*) as *n* → ∞. Additionally,

$$\begin{array}{c} L_{P}\left(\phi ,\mu ,\alpha |t\right)\to L\left(\phi ,\mu ,\alpha |t\right)\;\;\text{as}\;\;\;n\to \infty . \end{array}$$ (36)

Therefore, the PML estimators of *ϕ*, *μ* and *α* converge to the MLEs. Hence, under the same mild conditions of the MLEs, the PML estimators are asymptotically normally distributed with a joint trivariate normal distribution given by

$$\begin{array}{c} \left(\hat{\phi },\hat{\mu },\hat{\alpha }\right)∼N_{3}(\left(\phi ,\mu ,\alpha \right),I^{-1}\left(\phi ,\mu ,\alpha \right))\;\;\text{for}\;\;n\to \infty . \end{array}$$ (37)

It is important to point out that it is not simple to check the regularity conditions necessary to ensure asymptotically normal distribution (see Lehman [37], Theorem 5.1, page 463). In fact, Prentice [24] showed that the approximate normal distribution for *ϕ*, using the ML theory, could not be achieved even for sample sizes equal to or larger than 400. This result can also be extended to the PML theory, since the MLEs and PML estimators are asymptotically equivalent. In order to overcome this problem, for small sample sizes, we considered the bootstrap approach presented by DiCiccio and Efron [25] to construct improved confidence intervals based on the PML estimates.

### 3.4 Maximum product of spacings estimators

As an alternative to the ML estimation method, the maximum product of spacings (MPS) is a robust method for estimating the unknown parameters of continuous univariate distributions. Cheng and Amin [38, 39] introduced this method, which was independently developed by Ranneby [40] as an approximation to the Kullback-Leibler information measure. Moreover, Cheng and Amin [39] proved some desirable properties of the MPS estimators, such as asymptotic efficiency, invariance and, more importantly, the consistency of these estimators holds under more general conditions than for MLEs.

The uniform spacings of a random sample from the GG distribution are defined as

$$\begin{array}{c} \begin{array}{cc} D_{i}\left(\phi ,\mu ,\alpha \right)= & F(t_{\left(i\right)}|\phi ,\mu ,\alpha )-F(t_{\left(i-1\right)}|\phi ,\mu ,\alpha )=\frac{1}{\Gamma (\phi )}\int_{\mu^{\alpha }t_{\left(i-1\right)}^{\alpha }}^{\mu^{\alpha }t_{\left(i\right)}^{\alpha }}w^{\mu -1}e^{-w}dw, \end{array} \end{array}$$ (38)

for *i* = 1, 2, …, *n* + 1, where *t*_(*i*)_ is the *i*-th order statistics, *F*(*t*_(0)_|*ϕ*, *μ*, *α*) = 0 and *F*(*t*_(*n*+1)_|*ϕ*, *μ*, *α*) = 1. This implies that $\sum_{i=1}^{n+1}D_{i}\left(\phi ,\mu ,\alpha \right)=1$.

The MPS estimates, ${\hat{\phi }}_{\text{MPS}}$, ${\hat{\mu }}_{\text{MPS}}$ and ${\hat{\alpha }}_{\text{MPS}}$, are obtained by maximizing the geometric mean of the spacings

$$\begin{array}{c} G(\phi ,\mu ,\alpha )=[\prod_{i=1}^{n+1}D_{i}\left(\phi ,\mu ,\alpha \right){]}^{\frac{1}{n+1}} \end{array}$$ (39)

with respect to *ϕ*, *μ* and *α*. Or equivalently, by maximizing the logarithm of the geometric mean of sample spacings (39):

$$\begin{array}{c} H(\phi ,\mu ,\alpha )=\frac{1}{n+1}\sum_{i=1}^{n+1}\;\text{log}\;(D_{i}\left(\phi ,\mu ,\alpha \right)). \end{array}$$ (40)

Thus, ${\hat{\phi }}_{\text{MPS}}$, ${\hat{\mu }}_{\text{MPS}}$ and ${\hat{\alpha }}_{\text{MPS}}$ can be obtained by solving the nonlinear equations:

$$\begin{array}{c} \frac{1}{n+1}\sum_{i=1}^{n+1}\frac{1}{D_{i}\left(\phi ,\mu ,\alpha \right)}[\Delta_{j}\left(t_{\left(i\right)}|\phi ,\mu ,\alpha \right)-\Delta_{j}\left(t_{\left(i-1\right)}|\phi ,\mu ,\alpha \right)]=0,\;\;\;\text{for}\;\;\;j=1,2,3. \end{array}$$ (41)

In practice, one problem that may occur is the presence of ties due to multiple observations with the same value. In this case, if *t*_(*i*)_ = *t*_(*i*−1)_ for some *i* ∈ {1, 2, …, *n* + 1}, then *D*_*i*_(*ϕ*, *μ*, *α*) = *D*_*i*−1_(*ϕ*, *μ*, *α*) = 0. Thus, the MPS estimators are sensitive to closely-spaced observations, especially ties. Notice that

$$\begin{array}{c} \lim_{t_{\left(i-1\right)}\to t_{\left(i\right)}}D_{i}\left(\phi ,\mu ,\alpha \right)=\lim_{t_{\left(i-1\right)}\to t_{\left(i\right)}}\int_{t_{\left(i-1\right)}}^{t_{\left(i\right)}}f\left(t|\phi ,\mu ,\alpha \right)dt=f\left(t_{\left(i\right)}|\phi ,\mu ,\alpha \right). \end{array}$$ (42)

Hence, *D*_*i*_(*ϕ*, *μ*, *α*) should be replaced by the corresponding likelihood *L*(*ϕ*, *μ*, *α*|*t*_(*i*)_) = *f*(*t*_(*i*)_|*ϕ*, *μ*, *α*) when *t*_(*i*)_ = *t*_(*i*−1)_.

Cheng and Amin [38] presented a useful comparison between the MLEs and MPS estimators:

$$\begin{array}{c} \begin{array}{cc} \text{log}\;(D_{i}\left(\phi ,\mu ,\alpha \right)) & =\text{log}\;(\int_{t_{\left(i-1\right)}}^{t_{\left(i\right)}}f\left(t|\phi ,\mu ,\alpha \right)dt)\\ & =\text{log}\;(f\left(t_{\left(i\right)}|\phi ,\mu ,\alpha \right)\left(t_{\left(i\right)}-t_{\left(i-1\right)}\right))+R\left(t_{\left(i\right)},t_{\left(i-1\right)}|\phi ,\mu ,\alpha \right)\\ & =\text{log}\;(f(t_{\left(i\right)}|\phi ,\mu ,\alpha ))+\text{log}\;\left(t_{\left(i\right)}-t_{\left(i-1\right)}\right)+R\left(t_{\left(i\right)},t_{\left(i-1\right)}|\phi ,\mu ,\alpha \right), \end{array} \end{array}$$ (43)

where *R*(*t*_(*i*)_, *t*_(*i*−1)_|*ϕ*, *μ*, *α*) is essentially of order *O*(|*t*_(*i*)_ − *t*_(*i*−1)_)|) and |*t*_(*i*)_ − *t*_(*i*−1)_| → 0 in probability as *n* increases. For standard situations, log(*D*_*i*_(*ϕ*, *μ*, *α*)) is basically the same as log (*f*(*t*_(*i*)_|*ϕ*, *μ*, *α*)) with respect to *ϕ*, *μ* and *α*, except for a negligible number of terms. Therefore, the MLEs and the MPS estimators are asymptotically equal and have the same properties, i.e.

$$\begin{array}{c} \left({\hat{\phi }}_{\text{MPS}},{\hat{\mu }}_{\text{MPS}},{\hat{\alpha }}_{\text{MPS}}\right)∼N_{3}(\left(\phi ,\mu ,\alpha \right),I^{-1}\left(\phi ,\mu ,\alpha \right))\;\;\text{for}\;\;n\to \infty . \end{array}$$ (44)

### 3.5 Anderson-Darling estimators

Here, we present one type of minimum distance estimators (also referred to as the maximum goodness-of-fit estimators), which is based on the Anderson-Darling statistic and, due to this, is known as the Anderson-Darling (AD) estimator. The AD estimates, ${\hat{\phi }}_{\text{AD}}$, ${\hat{\mu }}_{\text{AD}}$ and ${\hat{\alpha }}_{\text{AD}}$, of the GG model parameters *ϕ*, *μ* and *α*, are obtained by minimizing, with respect to *ϕ*, *μ* and *α*, the function

$$\begin{array}{c} A\left(\phi ,\mu ,\alpha \right)=-n-\frac{1}{n}\sum_{i=1}^{n}(2i-1)\;[\;\text{log}\;(F(t_{\left(i\right)}|\phi ,\mu ,\alpha ))+\text{log}\;(S(t_{\left(n+1-i\right)}|\phi ,\mu ,\alpha ))\;]. \end{array}$$ (45)

These estimates can also be obtained by solving the nonlinear equations:

$$\begin{array}{c} \sum_{i=1}^{n}(2i-1)[\frac{\Delta_{j}(t_{\left(i\right)}|\phi ,\mu ,\alpha )}{F(t_{\left(i\right)}|\phi ,\mu ,\alpha )}-\frac{\Delta_{j}(t_{\left(n+1-i\right)}|\phi ,\mu ,\alpha )}{S(t_{\left(n+1-i\right)}|\phi ,\mu ,\alpha )}]=0,\;\;\;\text{for}\;\;\;j=1,2,3. \end{array}$$ (46)

Turning now to a modified version of the AD statistic, the right-tail Anderson-Darling (RAD) estimates, ${\hat{\phi }}_{\text{RAD}},{\hat{\mu }}_{\text{RAD}}$ and ${\hat{\alpha }}_{\text{RAD}}$, of the parameters *ϕ*, *μ* and *α*, are obtained by minimizing the function

$$\begin{array}{c} R\left(\phi ,\mu ,\alpha \right)=\frac{n}{2}-2\sum_{i=1}^{n}F(t_{\left(i\right)}|\phi ,\mu ,\alpha )-\frac{1}{n}\sum_{i=1}^{n}(2i-1)\;\text{log}\;(S(t_{\left(n+1-i\right)}|\phi ,\mu ,\alpha )) \end{array}$$ (47)

with respect to *ϕ*, *μ* and *α*. These estimates can also be obtained by solving the nonlinear equations:

$$\begin{array}{c} -2\sum_{i=1}^{n}\Delta_{j}(t_{\left(i\right)}|\phi ,\mu ,\alpha )+\frac{1}{n}\sum_{i=1}^{n}(2i-1)\frac{\Delta_{j}(t_{\left(n+1-i\right)}|\phi ,\mu ,\alpha )}{S(t_{\left(n+1-i\right)}|\phi ,\mu ,\alpha )}=0,\;\;\;\text{for}\;\;\;j=1,2,3, \end{array}$$ (48)

where Δ_*j*_ (⋅|*ϕ*, *μ*, *α*), *j* = 1, 2, 3, are given in (11).

## 4 Simulation

In this section, we show the results of a simulation study carried out to compare the efficiency of the different frequentist methods used for estimating the three parameters of the GG distribution. Considering the proposed estimators, the following procedure was adopted:

1. Generate *N* samples of size *n* from the GG(*ϕ*, *μ*, *α*) distribution and compute the $\hat{\theta }=\left({\hat{\theta }}_{1},{\hat{\theta }}_{2},{\hat{\theta }}_{3}\right)=\left(\hat{\phi },\hat{\mu },\hat{\alpha }\right)$ estimates using the MM, OLS, WLS, ML, PML, MPS, AD and RAD methods;
2. Using $\hat{\theta }$ and ***θ*** = (*θ*_1_, *θ*_2_, *θ*_3_) = (*ϕ*, *μ*, *α*), compute the bias, $\sum_{k=1}^{N}\frac{\left({\hat{\theta }}_{k,j}-\theta_{j}\right)}{N}$, and the mean square error (MSE), $\sum_{k=1}^{N}\frac{{\left({\hat{\theta }}_{k,j}-\theta_{j}\right)}^{2}}{N}$, where ${\hat{\theta }}_{k,j}$ denotes the estimate of *θ*_*j*_ obtained from sample *k*, for *k* = 1, 2, …, *N* and *j* = 1, 2, 3.

With this approach, the most efficient estimation method returns both bias and MSE closer to zero. The simulations were conducted using the R software [41]. For numerical optimization, we used the SANN algorithm, which was described in Section 2.1. Finally, the chosen values of the simulation parameters were: *N* = 20, 000, *n* = {20, 30, …, 300} and ***θ*** = {(0.5, 0.5, 3), (0.4, 1.5, 4)}. It is important to point out that the results of this simulation study were similar for other choices of ***θ***. Since in real applications it is difficult to obtain good initial values, we assumed that the initial values are random and were generated from a uniform distribution on the interval (0, 4). Therefore, we also expect to obtain good estimates regardless of the initial values.

The estimation procedures needed to be performed under the same conditions to make the comparison meaningful. However, for some particular samples and estimation methods, the numerical techniques failed in finding the parameter estimates. Thus, we present the observed proportion of failures/errors of each method in Tables 1 and 2.

**Table 1:** The proportion of errors of the numerical methods used for finding the estimates of *ϕ* = 0.5, *μ* = 0.5 and *α* = 3, for different sample sizes *n* and considering the following estimation procedures: MM, PML, ML, MPS, OLS, WLS, AD and RAD.

*n*	MM	PML	ML	MPS	OLS	WLS	AD	RAD
20	0.880	0.000	0.020	0.000	0.995	0.994	0.000	0.000
30	0.895	0.000	0.009	0.000	0.992	0.991	0.000	0.000
40	0.902	0.000	0.006	0.000	0.992	0.991	0.000	0.000
50	0.905	0.000	0.004	0.000	0.991	0.990	0.000	0.000
60	0.905	0.000	0.003	0.000	0.990	0.990	0.000	0.000
70	0.908	0.000	0.003	0.000	0.989	0.989	0.000	0.000
80	0.906	0.000	0.004	0.000	0.989	0.989	0.000	0.000
90	0.906	0.000	0.003	0.000	0.990	0.989	0.000	0.000
100	0.910	0.000	0.003	0.000	0.990	0.989	0.000	0.000
110	0.916	0.000	0.003	0.000	0.990	0.990	0.000	0.000
120	0.908	0.000	0.002	0.000	0.990	0.990	0.000	0.000
130	0.909	0.000	0.004	0.000	0.990	0.989	0.000	0.000
140	0.908	0.000	0.003	0.000	0.992	0.991	0.000	0.000
150	0.910	0.000	0.003	0.000	0.992	0.990	0.000	0.000
160	0.908	0.000	0.003	0.000	0.991	0.990	0.000	0.000
170	0.906	0.000	0.002	0.000	0.991	0.989	0.000	0.000
180	0.905	0.000	0.003	0.000	0.989	0.989	0.000	0.000
190	0.911	0.000	0.002	0.000	0.991	0.988	0.000	0.000
200	0.909	0.000	0.002	0.000	0.990	0.989	0.000	0.000
210	0.913	0.000	0.004	0.000	0.991	0.989	0.000	0.000
220	0.913	0.000	0.003	0.000	0.989	0.988	0.000	0.000
230	0.907	0.000	0.003	0.000	0.991	0.990	0.000	0.000
240	0.914	0.000	0.003	0.000	0.990	0.989	0.000	0.000
250	0.908	0.000	0.003	0.000	0.992	0.989	0.000	0.000
260	0.911	0.000	0.002	0.000	0.989	0.989	0.000	0.000
270	0.912	0.000	0.003	0.000	0.993	0.992	0.000	0.000
280	0.911	0.000	0.002	0.000	0.991	0.990	0.000	0.000
290	0.911	0.000	0.003	0.000	0.992	0.989	0.000	0.000
300	0.911	0.000	0.002	0.000	0.992	0.991	0.000	0.000

**Table 2:** The proportion of errors of the numerical methods used for finding the estimates of *ϕ* = 0.4, *μ* = 1.5 and *α* = 4, for different sample sizes *n* and considering the following estimation methods: MM, PML, ML, MPS, OLS, WLS, AD and RAD.

*n*	MM	PML	ML	MPS	OLS	WLS	AD	RAD
20	0.758	0.000	0.017	0.000	0.985	0.985	0.000	0.000
30	0.761	0.000	0.008	0.000	0.981	0.979	0.000	0.000
40	0.778	0.000	0.005	0.000	0.983	0.980	0.000	0.000
50	0.768	0.000	0.004	0.000	0.979	0.975	0.000	0.000
60	0.782	0.000	0.004	0.000	0.982	0.980	0.000	0.000
70	0.777	0.000	0.003	0.000	0.983	0.979	0.000	0.000
80	0.774	0.000	0.002	0.000	0.981	0.978	0.000	0.000
90	0.778	0.000	0.003	0.000	0.981	0.979	0.000	0.000
100	0.786	0.000	0.002	0.000	0.979	0.980	0.000	0.000
110	0.781	0.000	0.002	0.000	0.981	0.979	0.000	0.000
120	0.774	0.000	0.002	0.000	0.981	0.980	0.000	0.000
130	0.781	0.000	0.002	0.000	0.980	0.978	0.000	0.000
140	0.785	0.000	0.003	0.000	0.983	0.980	0.000	0.000
150	0.780	0.000	0.002	0.000	0.981	0.977	0.000	0.000
160	0.784	0.000	0.002	0.000	0.980	0.977	0.000	0.000
170	0.787	0.000	0.002	0.000	0.981	0.978	0.000	0.000
180	0.782	0.000	0.002	0.000	0.982	0.978	0.000	0.000
190	0.788	0.000	0.002	0.000	0.982	0.981	0.000	0.000
200	0.786	0.000	0.001	0.000	0.981	0.979	0.000	0.000
210	0.777	0.000	0.003	0.000	0.981	0.979	0.000	0.000
220	0.781	0.000	0.003	0.000	0.981	0.980	0.000	0.000
230	0.781	0.000	0.002	0.000	0.981	0.975	0.000	0.000
240	0.789	0.000	0.003	0.000	0.982	0.978	0.000	0.000
250	0.781	0.000	0.003	0.000	0.980	0.976	0.000	0.000
260	0.783	0.000	0.003	0.000	0.980	0.978	0.000	0.000
270	0.783	0.000	0.003	0.000	0.980	0.977	0.000	0.000
280	0.790	0.000	0.003	0.000	0.983	0.979	0.000	0.000
290	0.784	0.000	0.002	0.000	0.979	0.977	0.000	0.000
300	0.782	0.000	0.002	0.000	0.982	0.978	0.000	0.000

**Fig 2:**
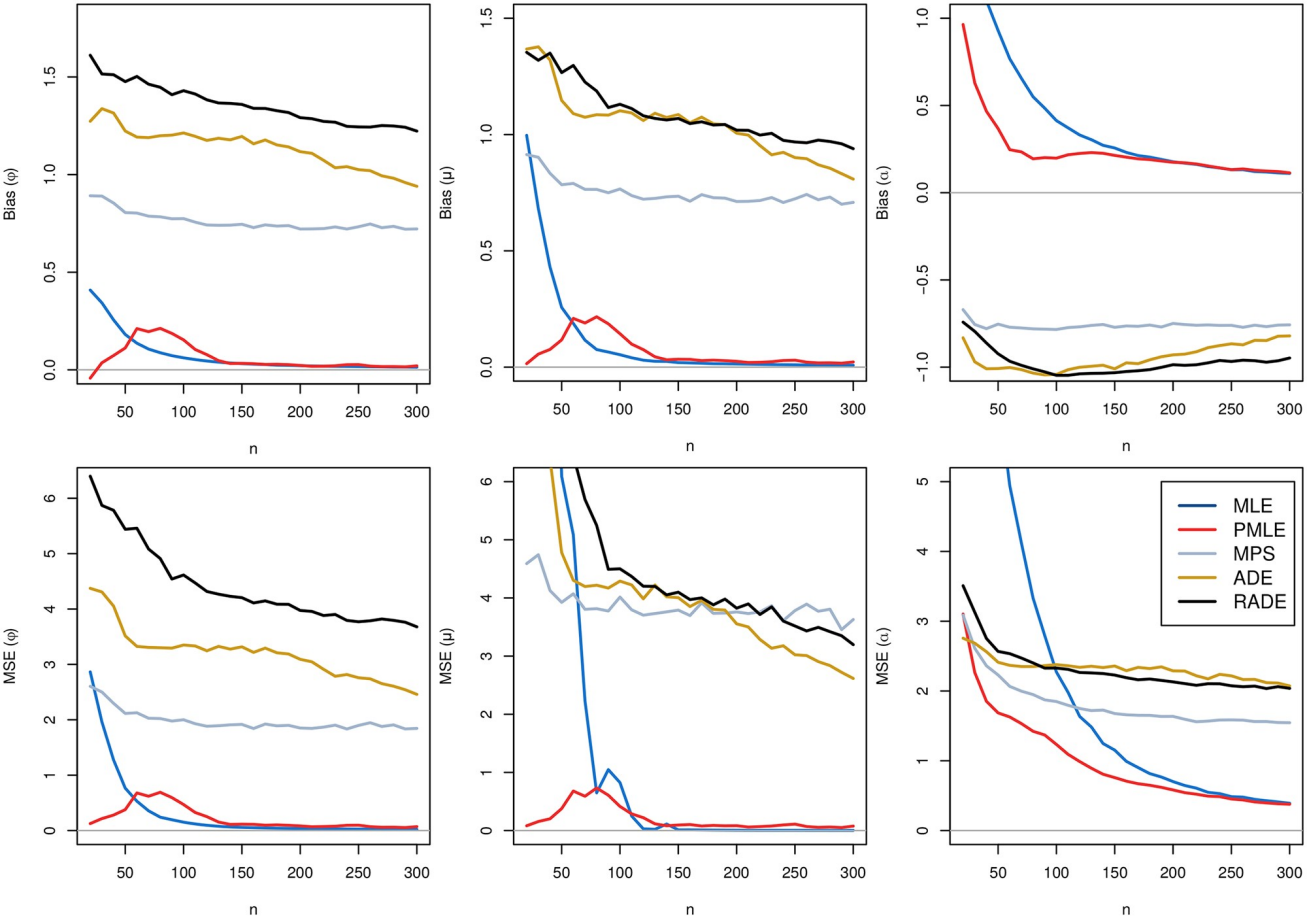
Comparison of the different estimation methods. Bias and MSE of the estimates of *ϕ* = 0.5, *μ* = 0.5 and *α* = 3, for *N* = 20, 000 simulated samples of size *n*, and using the following methods: 1-ML, 2-PML, 3-MPS, 4-AD, 5-RAD. The horizontal lines in these figures correspond to bias and MSE equal to zero. See text for explanations, definitions and notation.

**Fig 3:**
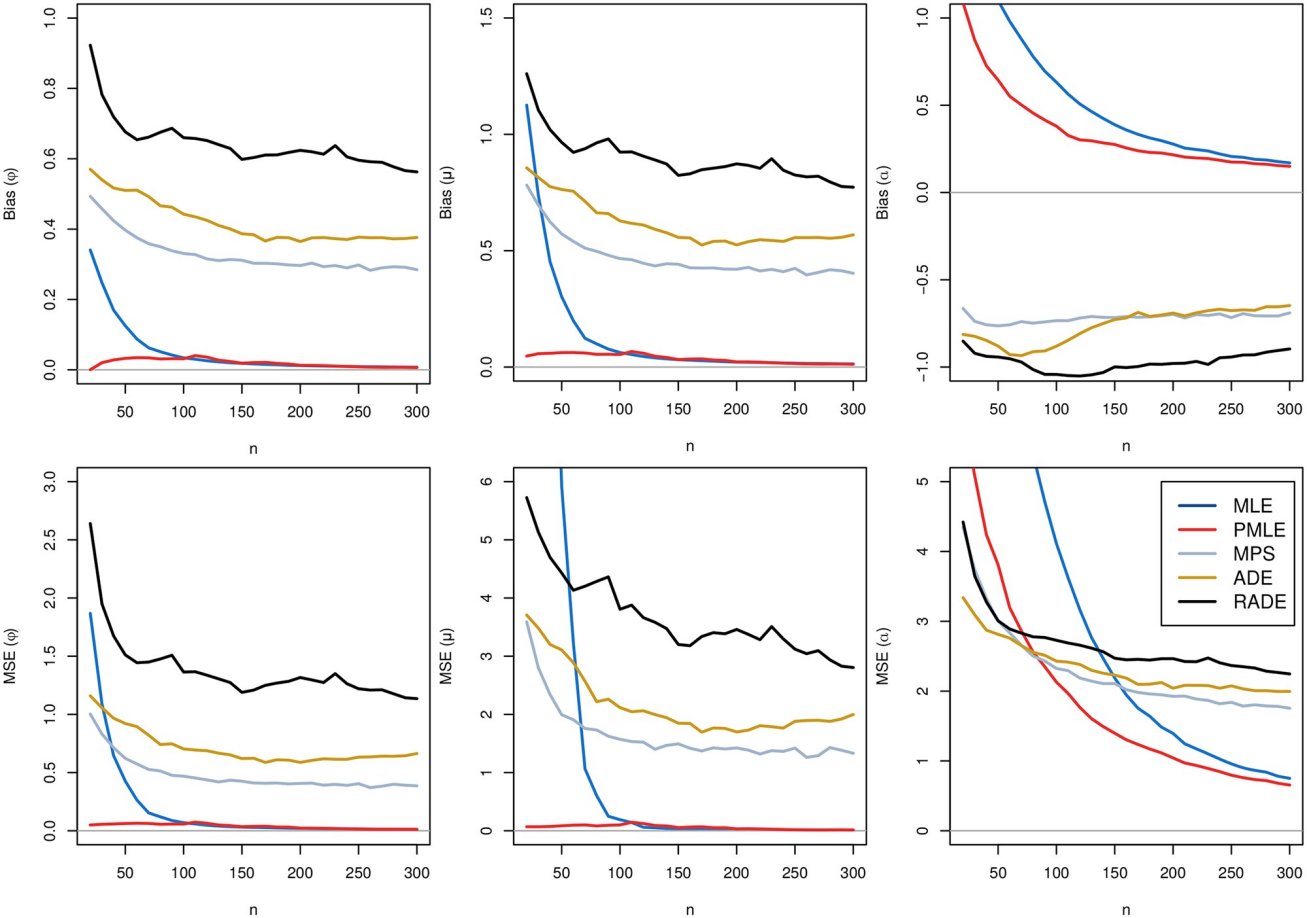
Comparison of the different estimation methods. Bias and MSE of the estimates of *ϕ* = 0.4, *μ* = 1.5 and *α* = 4, for *N* = 20, 000 simulated samples of size *n*, and using the following methods: 1-ML, 2-PML, 3-MPS, 4-AD, 5-RAD. The horizontal lines in these figures correspond to bias and MSE equal to zero. See text for explanations, definitions and notation.

As can be seen in these tables, there are high proportions of failures in the optimization process to find the estimates for the MM, OLS, and WLS methods, even for moderate sample sizes. Therefore, such estimation procedures should be avoided when estimating the parameters of the GG distribution. These methods were removed to avoid the inclusion of bias in the results to continue the simulation study. Hereafter, we consider the PML, ML, MPS, AD, and RAD estimators. Figs 2 and 3 present the bias and MSE of the estimates of *ϕ*, *μ* and *α*.

The horizontal lines in these figures correspond to bias and MSE equal to zero. In Figs 2 and 3, we observe that both the bias and MSE for all estimators tend to zero as *n* increases, i.e., the estimators are asymptotically unbiased and consistent for the parameters. The PML method returned improved estimates for the GG distribution when compared with the ML method. Moreover, the SANN algorithm allowed us to successfully find the estimates regardless of the initial values used for starting the optimization process. In this case, under the PML method, all the generated samples returned satisfactory estimates, even for small sample sizes. Therefore, combining all simulation results with the useful properties of the PML estimators, such as asymptotic efficiency, normality, consistency, and invariance, we conclude that the PML estimators should be used for estimating the parameters of the GG distribution.

## 5 Applications

We applied the proposed statistical methods to a data set of male patients admitted to the Emergency Department of the Ribeirão Preto Medical School, University of São Paulo, Brazil, diagnosed with TBI due to car accidents (excluding patients who where less than 18 years old, or other neurological conditions). Only patients that were admitted and discharged alive were considered in this study. Thus, we did not consider censored data. We considered the length of stay in hospital in the survival function. Our main aim here was to check the average time that a patient stays in hospital, given that some time had already passed. For example, if a patient has been in hospital for ten days, how much longer do we expect him/her to take to be discharged? This problem is discussed in this section.

The TBI data set was firstly studied by Tavares [42]. The data acquisition period was from May 2004 to June 2005, and the male patients were analyzed using three different lifetime variables: the total amount of time (in days) spent in hospital (hereafter, data set D1); the amount of time at the Neurology Inpatient Department (data set D2); and during which time patients used Mechanical Ventilation (data set D3).

We compared the results obtained using the GG distribution with the corresponding ones achieved with the usage of other three-parameter lifetime distributions. The Generalized Weibull (GW) distribution (Mudholkar et al. [43]), with pdf given by

$$\begin{array}{c} f\left(t|\lambda ,\alpha ,\sigma \right)={\left(\alpha \sigma \right)}^{-1}{\left(\frac{t}{\sigma }\right)}^{\frac{1}{\alpha }-1}{\left(1-\lambda {\left(\frac{t}{\sigma }\right)}^{\frac{1}{\alpha }}\right)}^{\frac{1}{\lambda }-1}, \end{array}$$ (49)

where λ∈R and *α* > 0 are the shape parameters and *σ* > 0 is the scale parameter. The Exponentiated Weibull (EW) distribution (Mudholkar et al. [44]), with pdf

$$\begin{array}{c} f\left(t|\theta ,\alpha ,\sigma \right)=\frac{\alpha \theta }{\sigma }{\left(\frac{t}{\sigma }\right)}^{\alpha -1}e^{-{\left(\frac{t}{\sigma }\right)}^{\alpha }}(1-e^{-{\left(\frac{t}{\sigma }\right)}^{\alpha }}{)}^{\theta -1}, \end{array}$$ (50)

where *θ* > 0 and *α* > 0 are the shape parameters and *σ* > 0 is the scale parameter. The Marshall-Olkin Weibull (MOW) distribution (Marshall and Olkin [45]), with pdf given by

$$\begin{array}{c} f\left(t|\alpha ,\beta ,\lambda \right)=\frac{\alpha \beta \lambda {\left(\lambda t\right)}^{\beta -1}e^{-{\left(\lambda t\right)}^{\beta }}}{{\left(1-\left(1-\alpha \right)e^{-{\left(\lambda t\right)}^{\beta }}\right)}^{2}}, \end{array}$$ (51)

where *α* > 0 and *β* > 0 are the shape parameters and λ > 0 is the scale parameter. Finally, the Extended Poisson-Weibull (EPW) distribution (Ramos et al. [46]), whose pdf is

$$\begin{array}{c} f\left(t|\lambda ,\alpha ,\beta \right)=\frac{\alpha \lambda \beta t^{\alpha -1}e^{-\beta t^{\alpha }-\lambda e^{-\beta t^{\alpha }}}}{1-e^{-\lambda }}, \end{array}$$ (52)

where $\lambda \in R^{*}$ and *α* > 0 are the shape parameters and *β* > 0 is the scale parameter.

The goodness-of-fit of the models was checked using the Kolmogorov-Smirnov (KS) test, which is based on the KS statistic: *D*_*n*_ = sup|*F*_*n*_(*t*) − *F*(*t*|***θ***)|, where sup is the supremum of the set of distances, *F*_*n*_(*t*) is the empirical cdf and *F*(*t*|***θ***) is the cdf of the reference distribution. The KS hypothesis testing was conducted at the 5% level of significance, to reveal whether or not the data came from *F*(*t*|***θ***). In this case, the null hypothesis (i.e. the data came from *F*(*t*|***θ***)) is rejected if the returned p-value is smaller than 0.05.

To carry out the model selection, the following discrimination criteria were adopted: AIC (Akaike Information Criterion) (Akaike [47]) and AICc (Corrected Akaike Information Criterion) (Sugiura [48]; Hurvich and Tsai [49]), which are computed by $\text{AIC}=-2\ell (\hat{\theta }|t)+2c$ and AICc = AIC + 2 *c* (*c* + 1)/(*n* − *c* − 1), where *c* is the number of model parameters and $\hat{\theta }$ is the estimate of ***θ***. Given a set of candidate models for the data at hand, the best fitted model is the one that presents the minimum values of these criteria. Furthermore, in order to distinguish between two almost equally well-fitting models, Burnham and Anderson [50], page 70, give a rough rule of thumb for comparing AICs (as well as AIC variations, including AICc), based on the AIC differences, Δ_*w*_ = AIC_*w*_ − AIC_min_, where AIC_*w*_ denotes the AIC value of the candidate model *w* and AIC_min_ is the minimum of the AIC values. Thus, models with Δ_*w*_ < 2 are all plausible, i.e. they have substantial support and should receive consideration in making inferences; models with 4 < Δ_*w*_ < 7 have considerably less support; and finally, models with Δ_*w*_ > 10 have either essentially no support and might be omitted from further consideration, or at least fail to explain some substantial explainable variation in the data.

Table 3 shows some summary statistics for these lifetime variables/data sets. According to all statistics, patients would spend less time, in days, on the Mechanical Ventilation than in the Neurology Inpatient Department.

**Table 3:** Summary statistics of patients’ TBI after a traffic accident, per data set. SD = Standard deviation, Min = Minimum, Max = Maximum.

Data Set	Mean	SD	Min	Median	Max	*n*
D1	27.105	19.061	4	23	67	19
D2	15.368	12.540	2	12	51	19
D3	10.875	8.310	1	10	29	16

As can be seen in Table 3, we have a small number of observations for the different data sets, which constitutes a situation where the ML method may return high-biased estimates. However, such a problem can be easily overcome by considering the PML estimators, as shown in Section 4. In TBI, specifically, the patient follow-up is time-consuming and requires dedication in order to acquire the data. Thus, small data sets may be recurring and solutions should be provided. Consequently, some models were fitted, and according to the model selection criteria, the best-adjusted one was indicated.

**Fig 4:**
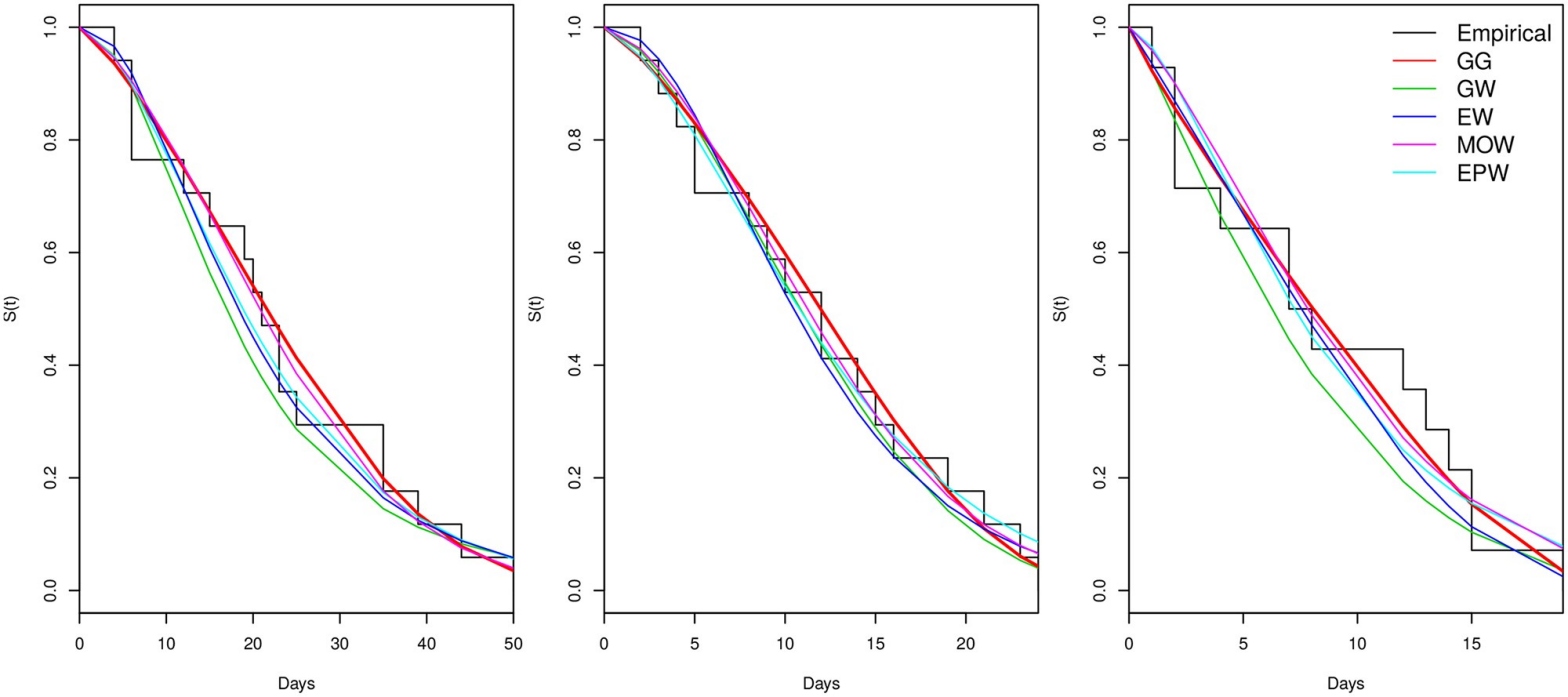
Data fitting. Survival functions superimposed to the empirical survival function, considering (A) D1 (B) D2 (C) D3 related to patients’ TBI caused by traffic accidents.

Table 4 provides the AIC and AICc values, as well as the p-values obtained from the KS test, for all five distributions (GG, GW, EW, MOW, and EPW) fitted using the PML estimation method. Observe that, for all three data sets, both criteria provide empirical evidence in favor of the GG distribution. However, the difference between the AIC (AICc) value for the EW model and the AIC (AICc) value for the GG model (“best” model according to both criteria) is less than two. Therefore, we can also consider the EW model as a plausible one for describing all the data sets. Followed up by Fig 4, which presents the fitted survival functions superimposed to the empirical survival function, it can be observed that the GG distribution gives a good fit to all data sets.

**Table 4:** The AIC, AICc and p-value from the KS goodness-of-fit test, for the fitted distributions, considering the three data sets related to patients’ TBI caused by traffic accident.

Data Set	Criterion	GG	GW	EW	MOW	EPW
D1	AIC	**139.912**	142.619	141.314	140.378	140.819
	AICc	**141.758**	144.466	143.160	142.224	142.665
	KS	0.9382	0.4206	0.7203	0.8910	0.8260
D2	AIC	**116.582**	116.627	118.087	117.417	117.916
	AICc	**118.429**	118.473	119.933	119.263	119.762
	KS	0.9554	0.9641	0.8989	0.9179	0.9936
D3	AIC	**89.915**	91.805	90.617	92.063	92.652
	AICc	**92.315**	94.205	93.017	94.463	95.052
	KS	0.9393	0.4228	0.7006	0.7144	0.7106

Obtained results support elements towards the development of a decision-making system, using ad hoc evidence, generating its associated probabilistic function, which helps the expert to infer patients’ risks. In addition to the point parameter estimates, we computed the confidence intervals, as well as the mean residual lifetime for the GG model parameters.

To construct such confidence intervals, one can use the asymptotic properties of the PML estimators. However, for the considered data sets, we have sample sizes smaller than 20. Prentice [24] showed that the approximate normal distribution for *ϕ*, using the ML theory, could not be achieved even for sample sizes equal to or larger than 400. Therefore, we considered a bootstrap approach to build such intervals (see DiCiccio and Efron [25]). It is essential to point out that the obtained bootstrap interval relies on a replication of small samples and the estimation of the related parameters, therefore the proposed approach that does not fail in finding such estimates plays an important role also in computing intervals. The PML estimates and the 95% bootstrap confidence intervals (CI) for the parameters *ϕ*, *μ* and *α* of the GG distribution, for all three data sets, are given in Table 5.

**Table 5:** PML estimates and 95% CI for the parameters of the GG distribution, per data set.

Data Set	*ϕ*	CI_95%_(*ϕ*)	*μ*	CI_95%_(*μ*)	*α*	CI_95%_(*α*)
D1	0.410	(0.248; 1.038)	0.025	(0.020; 0.040)	3.040	(1.831; 3.679)
D2	0.268	(0.178; 0.665)	0.045	(0.038; 0.063)	4.658	(2.753; 5.377)
D3	0.148	(0.100; 0.342)	0.053	(0.045; 0.074)	6.065	(4.094; 6.909)

Following the interpretation related to Table 5, data set D1 showed a higher estimate for the parameter *ϕ* than the others, where the parameters *μ* and *α* behave in the opposite way. It is worth mentioning that it is hard to obtain a biological interpretation for the parameters since they influence the higher moments (e.g., the mean and variance) of the distribution simultaneously. Moreover, the obtained bootstrap confidence intervals returned accurate evidence even considering small sample sizes.

From the proposed methodology, the PML estimates were obtained with a satisfactory goodness of fit. With the adjusted parameters, one can solve the problem related to the expected time that will be taken for a patient to be discharged. In order to achieve that, we consider the mean residual lifetime of the GG distribution, which is given by

$$\begin{array}{c} r\left(t|\phi ,\mu ,\alpha \right)=\frac{1}{S(t|\phi ,\mu ,\alpha )}\int_{t}^{\infty }wf\left(w|\phi ,\mu ,\alpha \right)dw-t=\frac{\Gamma (\phi +\frac{1}{\alpha },{\left(\mu t\right)}^{\alpha })}{\mu \Gamma (\phi ,{\left(\mu t\right)}^{\alpha })}-t. \end{array}$$ (53)

**Fig 5:**
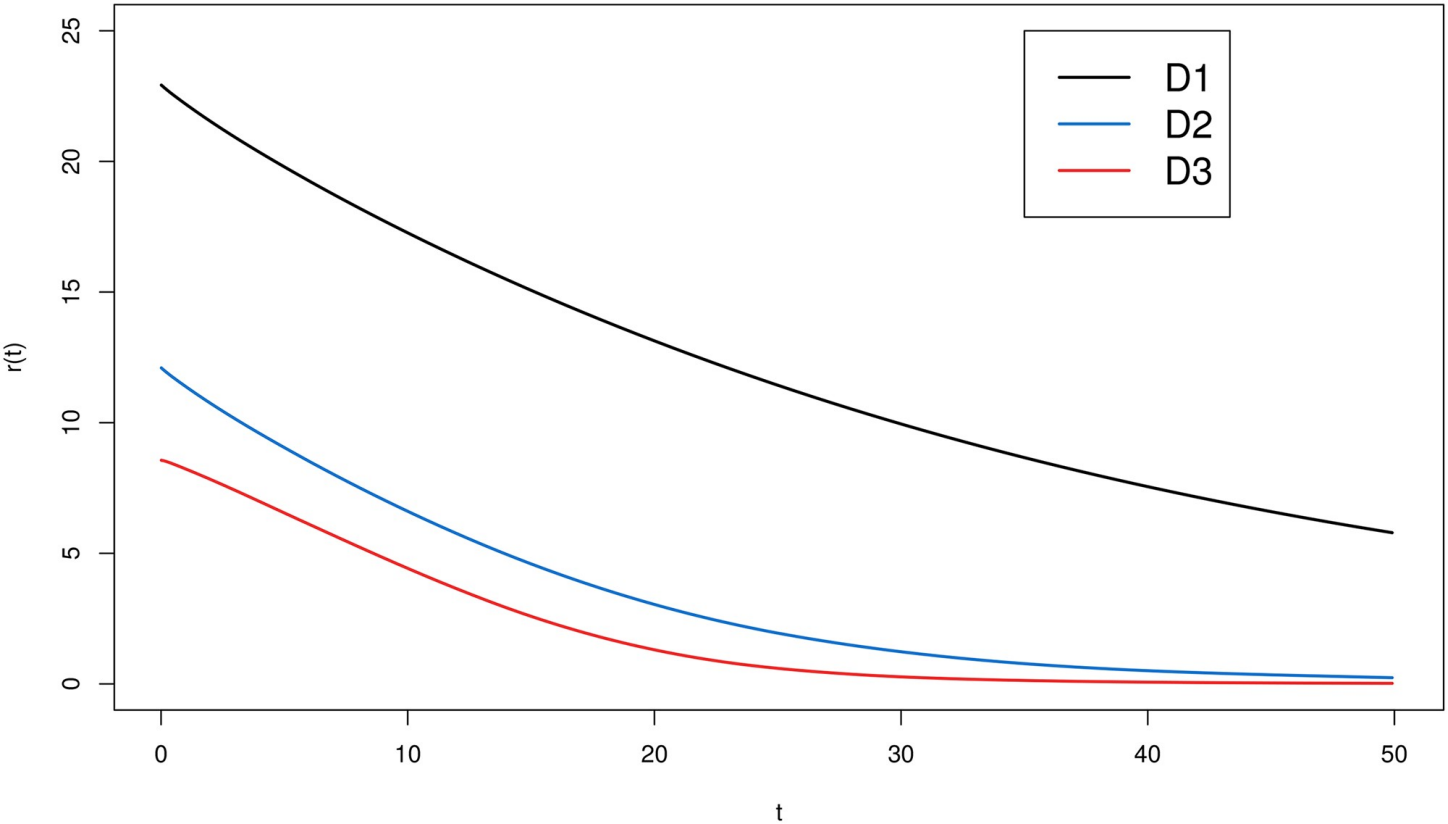
Mean residual lifetime. The estimation of the mean residual lifetime considering the three data sets related to patients’ TBI caused by traffic accidents.

Fig 5 shows the mean residual lifetime, considering the three data sets related to patients’ TBI caused by a traffic accident. The plotted curves return the conditional expectation (*r*(*t*)) given the patient’s spent time (*t*).

From this graph, we can see different expected times given that the patient has been in hospital. For instance, considering the patients from data set D1, given that one patient has been in hospital for ten days, we expect that he/she may be discharged after seventeen more days. On the other hand, if the patient is from data set D3 and has been hospitalized for ten days, then we expect that he/she will leave hospital after four more days.

## 6 Conclusions

In this paper, we derived and compared, through an extensive simulation study, different frequentist estimation methods for the parameters of the GG distribution. From our simulations, we observed that the OLS, WLS, and MM methods failed in finding the parameter estimates for a significant number of samples. On the other hand, considering the SANN algorithm with the PML estimation method, we were able to find the solutions (i.e., the parameter estimates) for all samples regardless of the initial values used for initiating the iterative procedure. Moreover, the PML method provided better estimates for all three parameters regardless of the sample size. Thus, the PML method is the most efficient estimation procedure, among the ones considered in this study, and should be used for all practical purposes.

Finally, our proposed methodology was illustrated in three real data sets related to patients’ traumatic brain injury caused by a traffic accident, demonstrating that the GG distribution is a simple alternative to be used in such applications for different occurrence rates and risks, even under the presence of small samples.

## Supporting information

S1 File Data are available downloading this file. (XLS)
